# A telomere-to-telomere gap-free genome of the new cultivar ‘Zhongtian No. 5’, combined with pan-genome analysis, aids in exploration and genetic enhancement of red clover (*Trifolium pratense* L.)

**DOI:** 10.1093/hr/uhag013

**Published:** 2026-01-13

**Authors:** Guangxin Cui, Chunmei Wang, Tianfen Guo, Fang Wu, Xia Wen, Xuehui Zhou, Biao Song, Jing Zhang, Xinqiang Zhu, Qian Zhang, Yuan Lu, Huirong Duan, Hongshan Yang

**Affiliations:** Lanzhou Institute of Husbandry and Pharmaceutical Sciences, Chinese Academy of Agricultural Sciences, Lanzhou, Gansu, China; Lanzhou Institute of Husbandry and Pharmaceutical Sciences, Chinese Academy of Agricultural Sciences, Lanzhou, Gansu, China; Laboratory of Quality & Safety Risk Assessment for Livestock Products, Ministry of Agriculture and Rural Affairs, Lanzhou Institute of Husbandry and Pharmaceutical Science, Chinese Academy of Agricultural Sciences, Lanzhou, Gansu, China; Lanzhou Institute of Husbandry and Pharmaceutical Sciences, Chinese Academy of Agricultural Sciences, Lanzhou, Gansu, China; Vocational Education Research Center，Yunnan Vocational College of Agriculture, Kunming, Yunnan, China; Lanzhou Institute of Husbandry and Pharmaceutical Sciences, Chinese Academy of Agricultural Sciences, Lanzhou, Gansu, China; Lanzhou Institute of Husbandry and Pharmaceutical Sciences, Chinese Academy of Agricultural Sciences, Lanzhou, Gansu, China; Lanzhou Institute of Husbandry and Pharmaceutical Sciences, Chinese Academy of Agricultural Sciences, Lanzhou, Gansu, China; Lanzhou Institute of Husbandry and Pharmaceutical Sciences, Chinese Academy of Agricultural Sciences, Lanzhou, Gansu, China; Lanzhou Institute of Husbandry and Pharmaceutical Sciences, Chinese Academy of Agricultural Sciences, Lanzhou, Gansu, China; Lanzhou Institute of Husbandry and Pharmaceutical Sciences, Chinese Academy of Agricultural Sciences, Lanzhou, Gansu, China; Lanzhou Institute of Husbandry and Pharmaceutical Sciences, Chinese Academy of Agricultural Sciences, Lanzhou, Gansu, China; Lanzhou Institute of Husbandry and Pharmaceutical Sciences, Chinese Academy of Agricultural Sciences, Lanzhou, Gansu, China

## Abstract

*Trifolium pratense* L. is a multifunctional crop of agronomic importance for forage, horticulture, and ecological restoration. However, the lack of a high-quality genome assembly and the limited representation of genetic diversity by a single reference have impeded its genetic research and molecular breeding. Here, we present the first telomere-to-telomere (T2T) gap-free genome for the diploid (2*n* = 2*x* = 14) cultivar *T. pratense* cv. ‘Zhongtian No. 5’ (TpraZt5), assembled through an integrated sequencing strategy. The 390.94 Mb assembly demonstrates high quality, with a base accuracy >98.5%, 98.1% Benchmarking Universal Single-Copy Orthologs (BUSCO) completeness, a long terminal repeat assembly index of 25.65, and a contig N50 of 52.95 Mb. We annotated 35 971 protein-coding genes and found repeat sequences accounting for 59.6% of the genome. The assembly resolved all seven centromeres and 14 telomeres, providing unprecedented insight into these complex genomic regions. We further constructed a 480.76 Mb pan-genome by integrating two additional accessions, which classified genes into core (70.2%), dispensable (25.3%), and private (4.5%) sets. Comparative genomic analyses identified 606 species-specific genes in TpraZt5 and uncovered extensive structural variations. Functional investigations revealed four species-specific genes and six contracted genes associated with isoflavonoid biosynthesis, two expanded chlorophyll a–b-binding proteins, and seven expanded auxin-related genes that may contribute to the high productivity of TpraZt5. Additionally, 44 *Gypsy*-type transposons within the zeatin biosynthesis pathway were identified as potential regulators of trifoliate leaf development. These genomic resources substantially improve structural annotation and functional characterization, providing vital tools for gene discovery and enhancing molecular breeding initiatives in red clover.

## Introduction

Red clover (*Trifolium pratense* L*.*) is not only a key temperate legume forage crop but also a valuable horticultural and ecological plant, cultivated across approximately 4 million hectares globally [1]. This versatile forage serves multiple agricultural functions including green manure application, temporary ground cover, landscaping, ecological restoration, sustainable gardening, and livestock feed through various utilization methods such as grazing, hay production, haylage, and silage. It also demonstrates remarkable agronomic advantages, including rapid establishment, shade tolerance, and adaptability to poorly drained, acidic soils. Its symbiotic nitrogen fixation capability significantly reduces dependence on synthetic nitrogen fertilizers, while its superior and stable protein content (nearly 20%) offers substantial potential for mitigating livestock production's environmental impact [2, 3]. When contrasted with alfalfa (*Medicago sativa*), red clover exhibits enhanced magnesium bioavailability (reducing grass tetany risk in grazing cattle) and improved postharvest protein preservation through unique biochemical mechanisms [2, 4]. Red clover biosynthesizes a broad spectrum of specialized secondary metabolites, particularly pharmacologically active flavonoids which demonstrate valuable therapeutic and nutraceutical applications [5, 6].

While contemporary breeding programs have successfully improved agronomic characteristics including stand persistence, pathogen resistance, and biomass production [1], significant opportunities remain for enhancing these traits along with other quality and nutritional parameters. Red clover is an allogamous diploid (2n = 2x = 14) species with high heterozygosity, which has hindered the assembly of a contiguous genome from short-read data. An effective gametophytic self-incompatibility system enforces strict outcrossing [7], with inbreeding depression precipitating a severe fitness loss by the S2–S3 generation [8]. These biological constraints have precluded the establishment of either stable inbred lines or doubled haploid populations, limiting functional genetic resources for this species [8]. The development of a high-quality reference genome would significantly advance genetic improvement in this species by enabling: accelerated genomic selection for agronomic and quality traits, enhanced physiological and biochemical characterization, and streamlined gene discovery pipelines.

The complexity of genomes, especially their abundant repetitive sequences, results in numerous gaps in reference genome assemblies. The emergence of third-generation sequencing (TGS) technologies has ushered in a transformative era for genome assembly. Notably, the integration of high-fidelity PacBio HiFi sequencing and ultra-long-read Oxford Nanopore Technologies (ONT) platforms has enabled unprecedented progress in resolving telomere-to-telomere (T2T) genomes. Within plant genomics, this technological leap has facilitated the successful assembly of T2T genomes for key model species, including *Arabidopsis thaliana*, *Oryza sativa*, and *Glycine max* [9–12]. To date, the number of published plant T2T genomes has expanded significantly, with dozens of studies documented and an accelerating publication trend [13]. These advancements underscore the pivotal role of T2T genomes as a foundational resource in modern genomics research. However, red clover still exhibits a substantial gap compared to model legumes in terms of assembly quality, data availability, and functional genomics applications, which hinders critical genomic analyses.

Recent studies have revealed substantial genomic variation among individuals within the same species, demonstrating that a single reference genome fails to capture the full spectrum of genetic diversity [14]. This critical insight has driven the progressive evolution and refinement of the pan-genome concept [15–20]. The pan-genome represents a complete genomic repertoire that encapsulates the collective genetic diversity within a species, consisting of core genes (genes present in all individuals), dispensable genes (genes present in two or more individuals), and private genes (genes present only in one individual). Genes governing biotic interactions, abiotic stress tolerance and phenotypic plasticity are commonly enriched in dispensable and private genes [21, 22]. Structural variations (SVs) are critical components of pan-genomic studies, which influence gene expression, 3D gene organization, and gene–gene interactions, thereby regulating multigene networks underlying complex traits [15, 23, 24]. Obviously, the resources of plant pan-genomes rather than single-reference genomes will accelerate molecular breeding [19, 25].

*Trifolium pratense* cv*. ‘*Zhongtian No. 5’ (TpraZt5) is a space-induced mutant cultivar developed by Lanzhou Institute of Husbandry and Pharmaceutical Sciences of Chinese Academy of Agricultural Sciences in Lanzhou, China. It was derived from the seeds of *T. pratense* cv*. ‘*Minshan’ that underwent space mutation aboard the Shenzhou-8 spacecraft. The cultivar was subsequently bred through successive rounds of selection and intercrossing within the mutated progeny to consolidate the stable five-leaflet phenotype. The new cultivar has growth and vegetative periods identical to Minshan (157 and 208 days, respectively) while exhibiting superior agronomic traits. Compared to Minshan, TpraZt5 exhibits a multifoliate rate of 79.2% (vs 0% for Minshan) with pentafoliolate leaves accounting for 78.9% of the total (vs 0% in Minshan). While the four-leaf clover is universally recognized as a symbol of good fortune, stable pentafoliolate variants are rare and regarded as botanical novelties. Within cultural semiotics, these atypical forms have acquired extended symbolic connotations, frequently representing concepts such as wealth, fame, and exceptional prosperity. Therefore, red clover populations producing tetra- or pentafoliolate leaves could serve as ornamental plants for believers. TpraZt5 exhibits a significantly larger leaf area (43.7 vs 27.9 cm^2^) with enhanced dry matter biomass (12 475 vs 10 757 kg/ha) and seed productivity (285 vs 231 kg/ha) compared to Minshan. Furthermore, it demonstrates superior nutritional and medicinal qualities, containing 0.4% total isoflavones and 20.2% crude protein, compared to 0.3% and 18.8% in Minshan, respectively. These enhanced characteristics make it an exceptional cultivar for both agricultural production and bioactive compound extraction. To comprehensively obtain its genetic information and gain deeper insights into its biological characteristics, we conducted whole-genome sequencing.

Here, we generated the T2T gap-free genome assembly of TpraZt5 by combining data from ONT, PacBio HiFi, Chromatin Conformation Capture (Hi-C) sequencing, and BGI bulk sequencing. Additionally, we performed a pan-genome analysis incorporating three red clover assemblies: the newly generated TpraZt5, along with previously released HEN17-A07 (TpraHA7) [26] and Milvus-BE (TpraMiv, GenBank assembly ID: GCA_949352195.3) genomes. These results elucidate the genetic basis of phenotypic variation among the three accessions, providing valuable genomic resources for targeted red clover breeding programs.

## Results

### Sequencing, assembly, and validation of T2T gap-free TpraZt5

**Figure 1:**
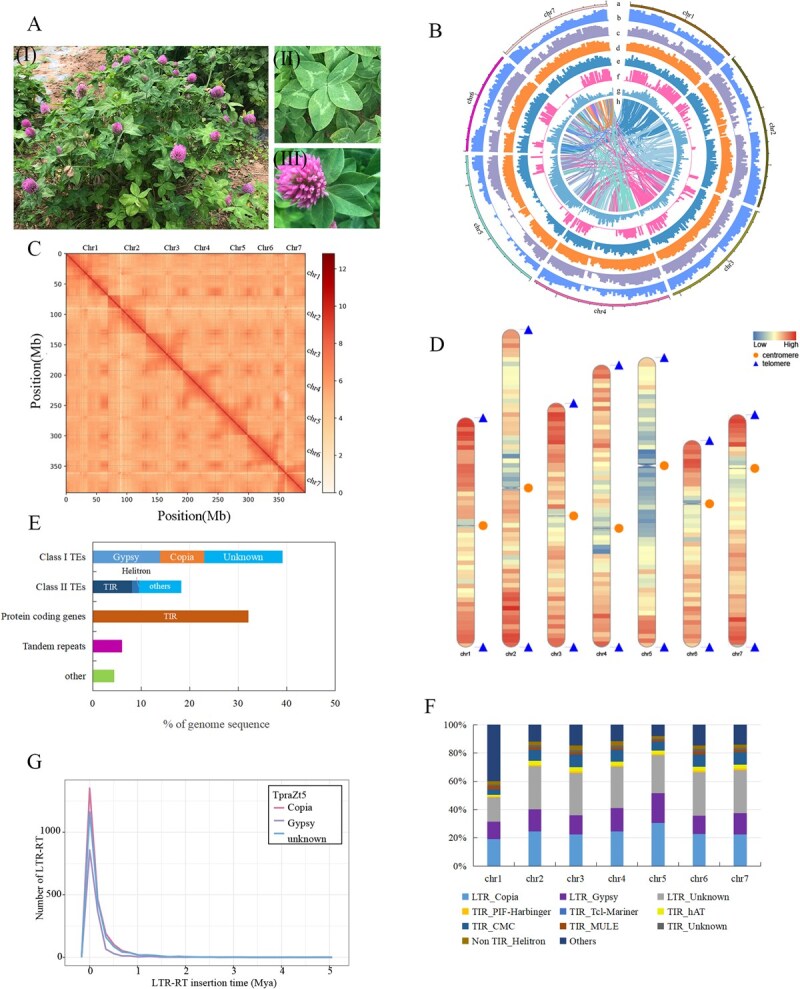
Genome assembly and genomic architecture of ZhongTian No. 5 (TpraZt5). (A) Morphology of TpraZt5. (I) A single plant. (II) A typical five-leaflet compound leaf. (III) A flower. (B) Circos diagram of major genomic characteristics of TpraZt5. The track from a to h is as follows: chromosomes, gene density (1 Mb window size), TE density (1 Mb window size), LTR density (1 Mb window size), DNA transposon density (1 Mb window size), Single nucleotide polymorphisms and insertions / deletions (SNPs / Indels) density (1 Mb window size), GC content (1 Mb window size), Collinear blocks within the TpraZt5 assembly. (C) Hi–C interactions between the seven chromosomes of TpraZt5. The color scale bar, ranging from light yellow to dark red, represents the frequency of Hi-C interaction links from low to high. (D) Identification of telomeres and centromeres on the seven chromosomes. Gene density is visualized along the chromosomes, with colors ranging from red (high density) to blue (low density). The orange circles and blue triangles mark the positions of centromeres and telomeres, respectively. (E) Summary of the genomic annotation. The ‘other’ category comprises the nonprotein-coding genes, unclassified repeat regions, and genomic regions that cannot be annotated as genes / repeats / nonprotein-coding genes. (F) Proportions of 11 repeat types in the seven pseudochromosomes respectively. (G) Insertion times of *Copia*, *Gypsy*, and LTR-RTs. Mya, million years ago.

The genome size of TpraZt5 was estimated to be 419.66 Mb through *k*-mer analysis (Fig. S1). A complete T2T gap-free genome assembly for TpraZt5 (Fig. 1A) was generated by integrating multiplatform sequencing data, including ONT reads, PacBio HiFi sequencing, and Hi-C analysis (Fig. S2). ONT sequencing yielded 67.15 Gb (~172×) of clean reads, PacBio HiFi sequencing yielded 25.80 Gb (~66×) of sequencing data, while Hi-C sequencing generated 196.42 Gb (~502×) of clean data that enabled chromosome-level assembly. This was complemented by BGI bulk RNA-seq for annotation support (Table S1).

Primary assembly using Hifiasm (v0.16) with hybrid PacBio HiFi and ONT ultralong reads generated 524 contigs spanning 435.75 Mb, exhibiting an N50 of 24.72 Mb. Hi-C data were subsequently employed for chromosome-scale scaffolding using the 3D-DNA pipeline (v2.1), which anchored 90.3% (393.59 Mb) of the assembled sequences into seven pseudochromosomes, represented by 41 scaffolded contigs with an N50 of 24.14 Mb.

Through systematic gap filling utilizing complementary PacBio HiFi and ONT ultralong sequencing data, we successfully constructed a complete, gap-free T2T genome assembly for TpraZt5. The final assembly comprised seven fully resolved pseudochromosomes spanning 390.94 Mb with an N50 of 52.95 Mb (Fig. 1B; Tables S2 and S3), matching the haploid chromosome number (*n* = 7) in red clover. Chromosomal architecture validation was performed through high-resolution Hi-C contact mapping, which demonstrated the expected antidiagonal interaction pattern characteristic of properly folded eukaryotic chromosomes (Fig. 1C).

A total of 14 telomeric candidate regions (seven chromosomal pairs) were identified using the canonical telomere repeat (CCCTAAA/TTTAGGG) as a query (Fig. 1D, Table S4). Centromeric regions were computationally predicted based on repeat density, the mapping results of repetitive elements and chromosomal analysis by StringDecomposer (v1.1.2). The seven centromeres exhibited considerable size variation, spanning 0.034–0.584 Mb with an average length of 0.210 Mb (Fig. 1D; Fig. S3, Table S5).

The complete T2T assembly of TpraZt5 exhibited outstanding quality metrics across multiple validation approaches. Structural evaluation through Hi-C analysis revealed proper chromosomal organization without detectable misassemblies. Read mapping analysis using BGI reads revealed a 98.5% alignment rate with an average sequencing depth of 118× and 98.4% of the genome achieving ≥20× coverage. Base-level accuracy was confirmed by a quality value (QV) of 38.03, while Benchmarking Universal Single-Copy Orthologs (BUSCO) demonstrated 98.1% completeness of conserved eukaryotic genes. Remarkably, the genome assembly achieved a long terminal repeat assembly index (LAI) score of 25.65, which not only qualifies for the gold standard classification (LAI ≥20) but also demonstrates exceptional completeness in long terminal repeat retrotransposon (LTR-RT) representation.

### Genome annotation

Repetitive sequences were 232.89 Mb, representing 59.6% of the TpraZt5 genome, primarily composed of transposable elements (TEs), especially LTR-RTs that accounted for 39.1% of the entire genome assembly (Table S6). DNA transposons accounted for 8.1% of the genome (Fig. 1E). Analysis of the LTR-RTs insertion timing revealed that bursts of different LTR-RT types including *Copia*, *Gypsy*, and unknown retrotransposons occurred 0.0087, 0.0069, and 0.0111 Mya, respectively (Fig. 1G).

The genome annotation of TpraZt5 predicted 35 971 protein-coding genes, demonstrating high completeness with a BUSCO score of 97.9% (Tables S7 and S8). Functional characterization revealed that 97.8% (35 179) of these genes could be assigned putative functions (Table S7). Furthermore, we identified a comprehensive repertoire of noncoding RNA complement comprising 917 rRNA, 877 tRNA, 580 snRNA, and 124 miRNA genes (Table S9), representing essential regulatory components of the genome.

### Potential influence of LTRs on nearby genes of TpraZt5 genome

Given the high abundance of LTRs in the TpraZt5 genome, we systematically investigated their *cis*-regulatory potential on neighboring gene elements. Genomic localization analysis identified 1843 protein-coding genes located within 1 kb of *Gypsy* and *Copia* LTR-RTs, with an additional 1150 genes residing adjacent to the unknown type (Figs S4A, S5A, and  S6A). Functional annotation through the Kyoto Encyclopedia of Genes and Genomes (KEGG) pathway enrichment revealed differential pathway associations among these gene cohorts. *Gypsy*-proximal genes exhibited significant enrichment in fundamental cellular processes including cell cycle regulation and programmed cell death pathways. In contrast, genes adjacent to *Copia* elements showed predominant involvement in metabolic pathways, particularly those governing glycan biosynthesis and metabolism as well as cofactor and vitamin metabolic processes. The unclassified LTR-associated gene set demonstrated enrichment in both cellular homeostasis pathways and energy metabolism modules (Figs S4B, S5B, and  S6B).

### Evolutionary and comparative genomic analysis

**Figure 2:**
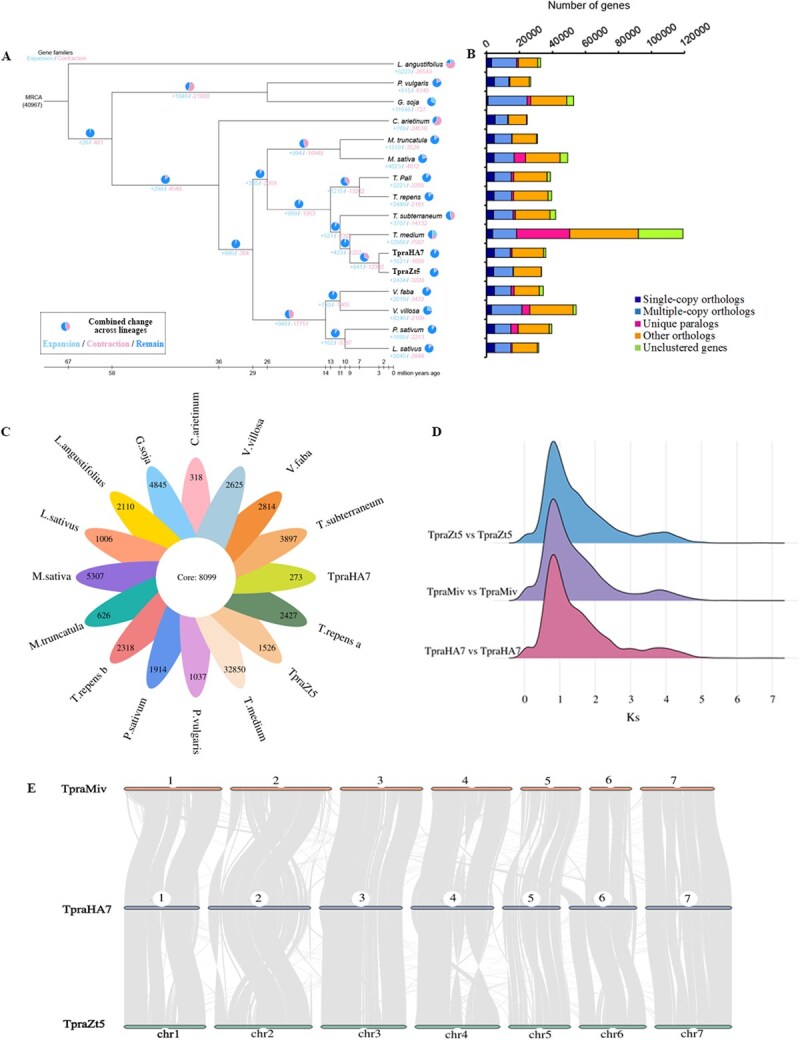
Comparative genomic analysis of the TpraZt5 T2T genome. (A) Phylogenomic relationships of TpraZt5 and related species inferred from single-copy orthologs. Gene family expansion / contraction counts are displayed at each node (positive numbers in sky blue indicate expansion; negative numbers in pale pink indicate contraction). The pie chart shows the proportion of gene families, with expansion in sky blue, contraction in pale pink, and stable families in blue. MRCA, most recent common ancestor. (B) Distribution of homologous gene categories shared by different species, including single-copy, multiple-copy, unique, unclustered, and other orthologs. (C) Venn diagram of orthogroups across the 15 analyzed species, including TpraZt5. (D) Synonymous substitution rate (*Ks*) density profiles of intraspecific comparisons of TpraZt5, HEN17-A07 (TpraHA7) [26] and Milvus-BE (TpraMiv, GenBank accession ID: GCA_949352195.3). The *Ks* peaks indicate ancient whole-genome duplication (WGD) events. (E) Synteny analysis among chromosomes of TpraZt5, TpraHA7, and TpraMiv. Gray lines connect syntenic genomic regions.

To elucidate the evolutionary history of TpraZt5, we conducted a comparative phylogenetic analysis with 14 other representative plant species, including five species from the *Trifolium* genus (Table S10). Using 92 conserved single-copy orthologs, we constructed a molecular phylogeny and estimated divergence times, revealing that TpraZt5 shares a closer evolutionary relationship with TpraHA7 than with *Trifolium medium*. Divergence time estimates indicated ~2 Mya since shared ancestry with TpraHA7 versus ~8 Mya for *T. medium* (Figs 2A and B).

Compared to the most recent common ancestor of TpraHA7, gene family analysis identified significant changes in the TpraZt5 genome, with 2434 expanded and 3228 contracted gene families (Fig. 2A). KEGG pathway enrichment analysis revealed significant functional divergence between expanded and contracted gene families in TpraZt5 (Figs S7 and S8; Tables S11 and S12). The expanded gene families demonstrated preferential enrichment (*P* <0 .05) in 44 functionally annotated pathways with distinct distribution patterns across five major categories: metabolic processes (40.9%), genetic information processing (22.7%), environmental information processing (15.9%), organismal systems (18.2%), and cellular processes (6.8%). Notably, two key biological pathways including plant hormone signal transduction and photosynthesis-antenna proteins exhibited particularly strong enrichment (Table S11). The hormone signal transduction pathway contained the largest number of expanded genes (92), predominantly including auxin-related genes such as auxin-induced proteins (15A, X10A, 6B, 10A5), auxin-responsive proteins (SAUR32, SAUR50, SAUR71), and L-tryptophan–pyruvate aminotransferase. In the photosynthesis-antenna proteins pathway, two crucial expanded genes were identified: chlorophyll a–b-binding proteins AB80 and 40, which play vital roles in light harvesting and energy transfer during photosynthesis. These findings suggest that gene family expansion in TpraZt5 has particularly enhanced its capabilities in hormone-mediated growth regulation and photosynthetic efficiency.

In contrast, contracted gene families were predominantly enriched (*P* <0 .05) in metabolic processes (49.2%) and organismal systems (26.2%), with particular significance observed in the isoflavonoid biosynthesis pathway (Table S12). This pathway exhibited contraction of six functionally annotated genes, including four putative isoflavone-7-O-methyltransferase homologs (rna-Tpr06578.1, rna-Tpr13589.1, rna-Tpr13576.1, rna-Tpr13577.1), one potential isoflavone 4′-O-methyltransferase gene (rna-Tpr13601.1), and one dihydroflavonol 4-reductase-like protein-coding gene (rna-Tpr29403.1). The contraction of these enzymatic components may lead to metabolic flexibility in isoflavonoid biosynthesis in TpraZt5 since they are critical for the structural modification and diversification of isoflavonoids.

Comparative genomic analysis revealed striking differences in gene family evolution between TpraZt5 and its relatives. While 8099 gene families were conserved across all 15 species, representing core ancestral functions, TpraZt5 exhibited pronounced lineage-specific innovation, with 1526 unique gene families (Fig. 2C). In contrast, TpraHA7 retained only 273 unique families. KEGG analysis showed that the unique genes in TpraZt5 were significantly enriched in the pathways ‘ubiquinone and other terpenoid-quinone biosynthesis’ and ‘motor proteins’ (Table S13). Whole-genome duplication (WGD) events in TpraZt5 were investigated through comparative genomic analyses with TpraHA7 and TpraMiv. Synonymous substitution rates (*Ks*) were calculated for homologous gene pairs to estimate the timing of duplication events. Two major peaks were observed in intraspecific comparisons, which implied at least two WGD events, with a more recent *Ks* peak (*Ks*: 0.81–0.83) than that shared with the three red clover cultivars (*Ks*: 3.80–3.95) (Fig. 2D). Furthermore, pairwise synteny analysis demonstrated strong macrosynteny between them, confirming their close relationship. However, large-scale genomic rearrangements could also be detected including inversion (INV), translocation (TRANS), and presence–absence variations (PAV), suggesting that large-scale changes followed the hybridization events or were shaped by differential selection pressures (Fig. 2E). These inferred WGD events and the overall collinearity patterns were further corroborated by synteny dot plot results (Fig. S9).

### Pan-genome analyses

**Figure 3:**
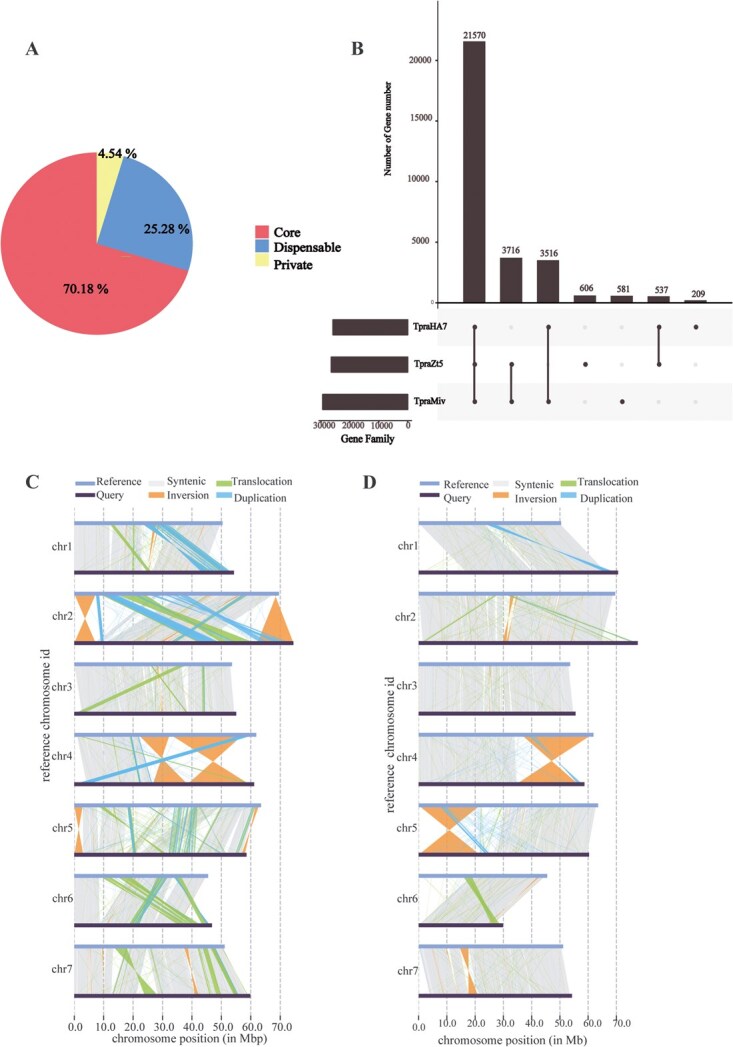
Pan-genome analysis and structural variations (SVs) of TpraZt5, HEN17-A07 (TpraHA7), and Milvus-BE (TpraMiv). (A) Proportions of core, dispensable, and private gene families in the assembled red clover pan-genome. (B) UpSet plot showing shared and unique gene families among TpraZt5, TpraHA7, and TpraMiv. (C–D) Structural rearrangements and synteny patterns in TpraHA7 (C) and TpraMiv (D) revealed by aligning each genome to the TpraZt5 T2T genome reference.

To investigate genetic variation and diversity across species and elucidate the genetic basis of phenotypic traits, we performed a pan-genome analysis of the *Trifolium* genus. This study utilized our newly assembled T2T genome of TpraZt5 as a reference, along with two publicly available chromosome-level genomes of TpraHA7 and TpraMiv. This pan-genome expanded the T2T TpraZt5 genome by adding 89.82 Mb. The analysis identified 21 570 core gene families (70.2% of the total), which were conserved across all accessions. Additionally, 7769 dispensable gene families (25.3%) were found in only two accessions, while a small proportion (4.5%) consisted of private gene sets unique to individual genomes (Fig. 3A). A higher degree of gene family conservation was observed between TpraZt5 and TpraHA7, with 25 286 shared orthologous gene families representing ~76.0% of the total gene repertoire in TpraHA7. In contrast, there were 22 187 shared orthologous gene families between TpraZt5 and TpraMiv, corresponding to merely 53.4% of the total gene content in TpraMiv. This disparity suggests a closer evolutionary relationship between TpraZt5 and TpraHA7 compared to TpraZt5 and TpraMiv (Figs 3B–D).

For the red clover pan-genome, the ratio of nonsynonymous to synonymous mutations was found to be higher in the dispensable and private gene set in comparison to the core gene set (Fig. S10A). In addition, the dispensable and private genes were found to be shorter on average than core genes (Fig. S10B). Comparative functional analysis revealed that core genes exhibited significantly higher evolutionary conservation and were predominantly enriched in essential cellular functions, such as cellular metabolism and translation (Figs S11A and S12A). In contrast, the dispensable and private gene sets showed specialized enrichment in secondary metabolic biosynthesis pathways that were associated with environment and defense responses; receptor and antioxidant activity (Figs S11B, C and S12B, C) such as phenylalanine, cutin, and suberine; ubiquinone and sesquiterpenoid biosynthesis in TpraZt5 (Fig. S12D); glucosinolate and betalain biosynthesis in TpraHA7; and diterpenoid biosynthesis in TpraMiv. These results are consistent with previous pan-genome studies in other angiosperms [21]. Of particular interest, TpraZt5 contains the largest complement of species-specific gene families (606); moreover, four distinct genes, rna-Tpr13038.1, rna-Tpr13040.1, rna-Tpr16090.1, and rna-Tpr16092.1, involved in isoflavonoid biosynthesis were identified (Table S14). This unique genetic repertoire suggests that TpraZt5 has evolved specialized metabolic capabilities, possibly reflecting adaptive evolution through secondary metabolite diversification.

### SV profiling of the assembled *Trifolium* pan-genome

**Figure 4:**
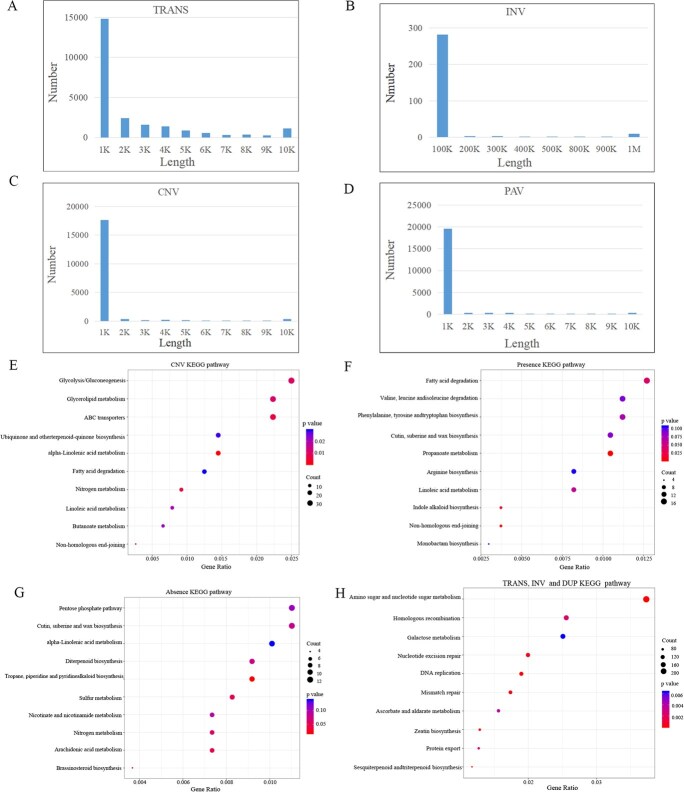
Characterization of SVs. (A–D) Length distributions of four major SV types: translocation (TRANS, A), inversion (INV, B), copy number variations (CNV, C), and presence–absence variations (PAV, D). (E–H) Top ten KEGG enrichment pathways for CNV (E), presence (F), absence (G), and other SVs including TRANS, INV, and DUP (H). Significant differences were assessed by a hypergeometric test (^*^FDR-adjusted *P* <0 .05).

Relative to the T2T reference genome of TpraZt5, SV profiling identified 58 862 and 63 395 SVs in TpraHA7 and TpraMiv, respectively. TpraMiv harbored a greater number of INV, TRANS, and copy number variations (CNV) including copy gain in nonreference genome (CPG) and copy loss in nonreference genome (CPL). Conversely, TpraHA7 exhibited a higher frequency of tandem duplications (DUP) than TpraMiv. Furthermore, PAV analysis identified 5138 presence and 5348 absence cases in TpraHA7, whereas TpraMiv contained 5337 presence and 5765 absence cases (Figs 3C and D; Table 1). TpraZt5 and TpraMiv exhibit extensive INV on chromosomes 2 and 4, as well as large-scale DUP on chromosomes 1 and 2. In contrast, comparisons between TpraZt5 and TpraHA7 revealed significant INV on chromosomes 4 and 5, but only minor DUP on chromosomes 1, 4, and 5 (Figs 3C and D). Among the total identified variations, ~62.3% of TRANS were concentrated within 1 kb and 10.1% were concentrated within 2 kb, INV (92.7%) were enriched in the size range of <100 kb, CNV (91.3%) were <1 kb and most PAV (90.7%) were <1 kb (Figs 4A–D).

**Table 1:** Comparative genomic structural variations of two cultivars using TpraZt5 as a reference

Type	TpraHA7	TpraMiv
TRANS	9765	13 954
INV	150	154
DUP	29 368	28 545
Presence number	5138	5337
Presence length (bp)	5 192 964	4 878 580
Absence number	5348	5765
Absence length (bp)	4 939 320	5 123 746
CPG	4432	4706
CPL	4661	4934

Presence, variants with a sequence length of >50 bp and not present in the reference genome are defined as existing variants; Absence, variants with a sequence length exceeding 50 bp that are present only in the reference genome are defined as deletion variants.

KEGG pathway enrichment analysis demonstrated significant associations between SVs and specific biological processes across the studied red clover accessions. Our analysis revealed that CNV were significantly enriched in α-linolenic acid metabolism pathway. PAV showed distinct enrichment patterns. Presence variants were significantly enriched in ‘propanoate metabolism’ and ‘nonhomologous end-joining’ while absence variants were significantly enriched in ‘tropane, piperidine, and pyridine alkaloid biosynthesis’, ‘arachidonic acid metabolism’, and ‘sulfur metabolism’ (Figs 4E–G). Notably, other SVs including DUP, INV, and TRANS exhibited significant enrichment in zeatin biosynthesis (Fig. 4H). Detailed examination of the zeatin biosynthesis pathway enables us to find that 80 candidate genes in TpraZt5 were clustered, with two dominant functional categories emerging: putative *Gypsy*-type transposon (*n* = 44), UDP-glucuronosyl and UDP-glucosyl transferase, which occupied 55% and 36% of the total genes, respectively (Table S15). Overall, our analyses provide a comprehensive catalog of SVs among the three accessions, supporting the study of SVs and will provide a prominent reference for the discovery of SVs in red clover populations.

## Discussion

### The T2T gap-free genome of TpraZt5 provides valuable genetic resources

Red clover is an exceptional horticultural, forage, and ecological crop due to its numerous agronomically advantageous traits [8, 26]. While conventional breeding strategies have achieved measurable gains in cultivar development, the integration of cutting-edge genomic tools now provides transformative potential for both trait discovery and breeding acceleration [1, 26]. Prior to this study, only 26 genome assemblies were available for *Trifolium* species, with varying levels of completeness including 15 chromosome-level, 6 scaffold-level, and 5 contig-level. The first genome assembly was scaffold-level (*Trifolium subterraneum*) [27], which was published in 2016, while nearly 70% of assemblies were released after 2020, with a notable surge in 2023–2024. As for *T. pratense*, NCBI currently hosts five genome records including three distinct accessions: Tatra [8], Milvus B [28] and Hen17-A07 [26]. Due to the species’ biological constraints including gametophytic self-incompatibility and inherent heterozygosity, existing short-read-based assemblies remain highly fragmented, with contig counts exceeding 135 000 (Table 2). These assemblies may exhibit significant scaffolding errors in both contig ordering and orientation. With the development of sequencing technology, genome assemblies have become increasingly better. The recent red clover HEN17-A07 genome assembly reported in 2022 [26] spanned 413.5 Mb and was composed of 319 contigs and 215 gaps. The other red clover Emerson genome reported by Yan *et al*. [29] was featured with the whole-assembly sequencing 423 Mb composed of 194 contigs, while the BUSCO completeness was 97.9%, which accounted for 92.8% of the predicted genome. The complete T2T gap-free genome assembly of TpraZt5 generated in this study represents a landmark achievement in *Trifolium* genomics, providing the most contiguous and comprehensively annotated reference genome for the genus to date. This milestone marks the entry of red clover into the T2T genomics era. The genome assembly demonstrates the highest quality reported to date, characterized by a gap-free status, an LAI score of 25.65, and 97.9% BUSCO completeness covering 97.8% of predicted genes. Leveraging this high-quality reference, we gained novel structural insights into previously challenging genomic regions such as centromeres, telomeres, and filled gaps.

Gene family analysis revealed that TpraZt5 has undergone significant expansion in genes encoding chlorophyll a–b-binding proteins (AB80 and 40) and auxin-related proteins compared to its most recent common ancestor with TpraHA7. Notably, the expansion was most pronounced within the auxin-responsive gene category. As integral components of thylakoid membranes in both photosystem I and II, light-harvesting chlorophyll a/b-binding proteins have been well documented to regulate agronomic traits in barley [30], modulate chlorophyll a biosynthesis in kiwifruit [31] and leaf color in albino tea germplasm [32], determine yield potential in rice [33] and oil palm [34], and mediate responses to various abiotic stresses [35–37]. Auxin is widely distributed in various parts of plants and participates in regulating growth, organ development, and morphogenesis [38]. Auxin/indole-3-acetic acid (Aux/IAA) and small auxin-up RNA (SAUR) are two major classes of auxin early response genes [39]. In the expanded gene sets of TpraZt5, we identified genes encoding auxin-induced proteins (15A, X10A, 6B, 10A5) of the Aux/IAA family, as well as genes encoding auxin-responsive proteins (SAUR32, SAUR50, and SAUR71) of the SAUR family. The Aux/IAA protein family functions as transcriptional repressors and plays a central regulatory role in auxin signal transduction [40, 41]. The functional roles of Aux/IAA genes have been demonstrated across species, including their involvement in apple fruit development [42]; the regulation of yield-related traits through grain length and leaf angle in rice [43]; and the promotion of plant height and lateral root initiation in transgenic tomato overexpressing peach *PpIAA19* [44]. SAUR genes have been reported to regulate diverse biological processes [45]. For instance, in *Arabidopsis*, SAUR32 modulates apical hook formation [46], while SAUR50 stimulates cell expansion during apical development and regulates overall plant growth [47]. Collectively, the significant expansion of key genes encoding chlorophyll a–b-binding proteins, Aux/IAA, and SAUR proteins is proposed as a putative driver for the higher biomass and seed productivity of TpraZt5 relative to its ancestor cultivar Minshan. This enhanced agronomic performance, potentially mediated by improved growth regulation and photosynthetic efficiency, requires validation in future studies.

**Table 2:** Genome assembly quality metrics comparison between TpraZt5 and five published reference genomes

Metrics	TpraZt5	HEN17-A07	Milvus_BE	Milvus	Milvus B	Tatra
GeneBank accession ID	PRJNA- 1332894	GCF_ 020283565	GCA_ 949352195	GCA_ 900292005	GCA_ 900079335	GCA_ 000583005
Assembly level	T2T	C	C	C	C	Contig
Number of contigs	7	319	911	135 014	135 023	267 372
Genome size (Mb)	390.9	413.6	445.6	351.6	346	305
Number of gaps	0	215	53	70 499	49 797	
Total unmapped length (Mb)	390.9	413.5	445.5	309	309	
Telomere number	14	4	5	0	0	
Expected telomere number	14	14	14	14	14	14
LAI	25.65	25.58	21.27			
N-base count (Mb)	0	0.11	0.005	37.32	24.96	0.066

‘C’, Chromosome; LAI, long terminal repeat assembly index; N-base count, count of undetermined nucleotides.

### Pan-genome analysis further facilitates the genetic dissection of agronomic traits in TpraZt5

The extensive genomic heterogeneity observed among conspecific individuals has revealed the inherent constraints of relying on a single-reference genome to capture intraspecies genetic diversity [14, 48]. Pan-genomes demonstrate superior capability in detecting SVs and mitigating reference bias compared to a single-reference genome [49–50]. The ongoing refinement of sequencing technologies and genome assembly methodologies, accompanied by progressively reduced sequencing costs, has accelerated the transition from single-reference approaches to pan-genome frameworks. This emerging paradigm offers a more comprehensive basis for studying evolutionary mechanisms, selective constraints, and gene functions, positioning pan-genomes as indispensable genomic resources for modern biological and agricultural research [15].

For *T. pratense*, due to the small size of the research community and its genome complexity, pan-genome-assisted breeding efforts remain limited. This study establishes the first pan-genome framework for red clover, filling a critical knowledge gap in genomic research and precision breeding. The private gene set significantly contributes to species diversity and environmental adaptation, serving as a major source of variation for agronomically important traits. For TpraZt5, we identified 606 private gene families, including four candidate genes involved in isoflavonoid biosynthesis that were classified into two functional categories: (i) rna-Tpr13038.1 and rna-Tpr13040.1, encoding putative isoflavone 2′-hydroxylase-like cytochrome P450 monooxygenases (CYP81E subfamily) that may catalyze the 2′-hydroxylation of isoflavone substrates and (ii) rna-Tpr16090.1 and rna-Tpr16092.1, encoding cytochrome P450 family 71 proteins (CYP71 clan) potentially involved in oxidative modifications during isoflavonoid biosynthesis. Moreover, in the contracted gene families of TpraZt5, six functionally annotated genes were identified including four putative isoflavone-7-O-methyltransferase homologs (rna-Tpr06578.1, rna-Tpr13589.1, rna-Tpr13576.1, rna-Tpr13577.1), one potential isoflavone 4′-O-methyltransferase gene (rna-Tpr13601.1), and one dihydroflavonol 4-reductase-like protein-coding gene (rna-Tpr29403.1). The contraction of these enzymatic components, combined with the four private genes described above, may play crucial roles in isoflavonoid metabolism in TpraZt5 and provide a genetic basis for explaining its higher isoflavonoid content compared to its ancestor cultivar Minshan.

Pan-genomic research has provided unprecedented insights into the functional significance of SVs as key determinants of phenotypic diversity. Empirical evidence reveals that SVs contribute substantially to: (i) environmental adaptation through modulation of abiotic stress responses, e.g. drought and salinity tolerance [51, 52] and pathogen resistance mechanisms [53, 54]; (ii) developmental regulation, particularly in photoperiod-dependent flowering time control [55]; and (iii) domestication syndrome traits including seed dispersal mechanisms [56] and plant architecture [57, 58]. A notable case study in pearl millet has further elucidated the association between specific SVs and transcriptional regulation of heat-responsive genes [59]. Therefore, systematic identification and characterization of plant SVs will facilitate in-depth investigations into genotype–phenotype associations, thereby promoting the development of more precise and efficient crop genetic improvement technologies.

The nonarticulate trifoliolate leaf configuration, characterized by leaflets attached to an extended petiole, constitutes the principal foliar morphology within the genus *Trifolium*. Documented instances of structural transition from trifoliolate to multifoliolate organization have been reported in *T*. *pratense*, *Trifolium repens*, and *Trifolium alexandrinum* [60]. A tetrafoliolate white clover genotype, demonstrating ~60% heritable expressivity, was engineered through mutagenic intervention [61]. Similar multifoliolate manifestations have been documented across various leguminous taxa, including *Arachis hypogaea* [62] and *Vigna radiata* [63]. The multifoliolate phenotype is postulated to significantly enhance biomass production through augmented photosynthetic surface area [64, 65], elevated protein content, and other improved qualitative attributes [66]. Nevertheless, the genetic underpinnings of the multifoliolate trait in the *Trifolium* genus remain elusive. Current evidence suggests that the *Trifolium* genus evolved from multifoliolate ancestral forms, with subsequent evolutionary reduction in leaflet number [67]. Genetic studies indicate that the multifoliolate trait in red clover (*T. pratense*) may be preconditioned by homozygous recessive alleles at one or two loci [68], or be governed by at least three additive recessive pairs of alleles, together with some modifying genes of incomplete penetrance [69], or determined by a quantitative recessive trait [70]. Furthermore, non-Mendelian inheritance characterized by the discrete nature of leaflet numeration, coupled with the occurrence of multifoliolate structures on individual branches and reversible foliar plasticity [40], has constrained the deciphering of the multifoliolate trait in *Trifolium*. Beyond these, the absence of stable breeding lines exhibiting nontrifoliolate leaf morphologies substantially increased the genetic investigation complexity. Heteroblasty, a potential adaptive strategy employed by plants to respond to heterogeneous environmental conditions—analogous to phenotypic plasticity—may account for the occurrence of chimeric plants [71, 72], but fails to explain the development of true-breeding pentafoliolate plants and their progeny exhibiting complete pentafoliolate leaf expression. Malaviya *et al*. postulated that TEs, operating in concert with epigenetic regulatory mechanisms, are likely instrumental in modulating leaflet numeration expression. Among the materials investigated in our study, most plants of the accessions TpraHA7 and TpraMiv exhibit regular trifoliolate leaves, while our TpraZt5 cultivar demonstrated a high five-leaflet rate of 78.9%. Notably, structural variant analysis of the constructed pan-genome revealed 44 *Gypsy*-type transposons embedded within the zeatin biosynthesis pathway, suggesting their potential involvement in regulating trifoliate leaf morphogenesis through DUP, INV, and TRANS events. This groundbreaking discovery provides pivotal genetic resources for elucidating the molecular mechanisms underlying leaf development in *Trifolium* species, revealing a novel regulatory paradigm wherein TEs interact with phytohormonal pathways to modulate leaf architecture.

This study presents the first T2T gap-free genome and the inaugural pan-genome of red clover, marking a seminal advance in its genomic characterization. Our analysis has identified an extensive set of candidate genes implicated in isoflavonoid biosynthesis, leaf morphogenesis, and yield-related traits. Future investigations should prioritize functional validation of these candidates and systematic elucidation of their biological mechanisms, thereby accelerating genetic improvement of red clover. However, constrained sampling limits the pan-genome’s representativeness, resulting in deficient genetic diversity capture and an inflated core gene set relative to those derived from extensive germplasm collections. Consequently, this limited assembly fails to reveal the extensive repertoire of unique sequences and SVs accessible through broader sampling. To fully exploit pan-genomic resources for red clover breeding, future efforts should incorporate broader germplasm collections, including integration of wild relatives to identify wild alleles that govern critical fitness traits.

## Materials and methods

### Plant materials

Healthy leaves were collected from TpraZt5 plants grown at the Lanzhou Scientific Observation and Experiment Field Station of the Ministry of Agriculture for Ecological Systems in the Loess Plateau area (36°10′N, 103°45′E, altitude 1700 m). The freshly harvested samples were immediately flash-frozen in liquid nitrogen. High-quality and high-molecular weight genomic DNA was extracted from the frozen leaves using the cetyltrimethylammonium bromide (CTAB) method [73]. The purity, integrity, and concentration of the extracted DNA were assessed by 1% agarose gel electrophoresis and quantified using a Qubit fluorimeter (Invitrogen, Carlsbad, CA, USA). This high-quality genomic DNA was subsequently used for PacBio, Nanopore, and BGI sequencing.

### Genome sequencing

High-quality genomic DNA was extracted and purified using the DNAsecure Plant Kit (TIANGEN, China). For PacBio HiFi sequencing, a size-selected (15–20 kb) SMRTbell library was prepared and sequenced on the PacBio Revio platform, generating high-fidelity circular consensus sequencing (CCS) reads for downstream analysis. For ultra-long-read sequencing, libraries were constructed using the Oxford Nanopore SQK-LSK109 kit and sequenced on a PromethION platform (Oxford Nanopore Technologies, UK). For Hi-C sequencing, young shoot tissues were fixed with formaldehyde, lysed, and digested with the MboI restriction enzyme. The resulting Hi-C libraries were then sequenced in 150 bp paired-end mode on the BGI DNBSEQ-T7 platform (BGI, China).

### Genome survey, assembly, and assessment

The genome size of TpraZt5 was estimated using a *k*-mer-based approach [74]. PacBio HiFi CCS reads were generated by processing raw HiFi reads using the CCS software (v6.0.0). For Nanopore sequencing, ultra-long reads were quality-filtered using Filtlong (v0.2.4) and adapter-trimmed with Porechop (v0.2.4) to retain ‘pass’ reads. To leverage the complementary strengths of PacBio HiFi reads (high accuracy) and Nanopore reads (long-range continuity), we performed a hybrid *de novo* assembly using Hifiasm (v0.15.1-r334) [75] with default parameters. Hi-C data were processed with Juicer (v1.6) [76] to align reads to the draft contigs. Chromosome-scale scaffolding was achieved using 3D-DNA [77], which clustered, ordered, and oriented the contigs based on chromatin interaction patterns. Manual curation was conducted in Juicebox (v2.13.07) to refine chromosomal boundaries and correct misassemblies. The curated assembly resolved seven chromosomes alongside unanchored contigs, representing a high-confidence reference genome.

To resolve remaining gaps in the genome assembly, we performed targeted gap filling using both PacBio HiFi CCS reads and Nanopore pass reads. These reads were aligned to the gap regions using Winnowmap (v1.11; parameters: *k* = 15, -MD) [78]. For each gap, we selected the longest high-quality alignment that spanned both gap ends for precise gap closure. Telomeric regions were systematically identified by screening for the canonical seven-base telomere repeats (CCCTAAA/TTTAGGG) using Nucmer (v3.1). Chromosomes lacking terminal telomeres were manually completed by patching these conserved repeat sequences. The gap-filled assembly underwent six rounds of iterative error correction using Racon (v1.6.0) [79], incorporating both HiFi long-read and short-read NGS data with default parameters. This rigorous polishing process yielded a complete T2T genome assembly for TpraZt5.

We performed comprehensive quality assessment of the TpraZt5 genome assembly through multiple approaches: Hi-C reads mapping ratios were calculated using BWA (v0.7.17-r1188) [80] to evaluate assembly continuity at the chromosomal scale; gene space completeness was assessed with BUSCO (v5.2.2) [81] against the Embryophyta-odb10 database; LTR-RT were accurately identified using LTR Retriever (v2.9.0) [82] to characterize repetitive elements; assembly accuracy was quantified through Merqury (v1.1) [83] analysis incorporating Hi-C, ONT, and HiFi reads; and potential structural errors were detected using CRAQ (v1.0.9) to validate assembly integrity, collectively ensuring the production of a high-quality reference genome suitable for downstream genomic analyses.

### Genome annotation

Repetitive sequences were identified in the genome assembly through a dual-strategy approach integrating both homology-based prediction and *ab initio* identification methods [84, 85]. Initial annotation of transposable elements was performed using RepeatMasker (v4.0.9) and RepeatProteinMask (v4.0.9) [86] against a curated repeat library incorporating known elements from the Repbase database [87]. To comprehensively identify novel repetitive sequences, a *de novo* repeat library was constructed utilizing RepeatModeler (v1.0.11), which integrates RepeatScout (v1.0.5) for identification of high-copy number repeats and LTR-FINDER (v1.0.5) [88] for specific detection of LTR-RTs. Furthermore, tandem repeat sequences were precisely annotated using Tandem Repeats Finder (TRF, v4.09) [89] with default parameters, enabling complete characterization of all major classes of repetitive elements within the genome assembly.

An integrative approach combining multiple evidence types was used to predict protein-coding genes in the TpraZt5 genome. Gene prediction was performed through three complementary strategies [90]: (i) homology-based prediction using protein sequences from three closely related species (*T. pratense*, *T. medium*, *T. repens*) and universal Swiss-Prot proteins, implemented with Exonerate (v2.2.0); (ii) *ab initio* prediction using Augustus (v3.3), Genscan (v1.0), and GlimmerHMM (v3.0.4) [91]; and (iii) transcriptome-based prediction through *de novo* assembly of RNA-seq data obtained from an integrated sample of roots, stems, leaves, flowers, and seeds of TpraZt5 sequenced on the BGI DNBSEQ-T7 platform (BGI, China), using StringTie (v2.1.1) [92] followed by genome alignment with GMAP (2020-10-24). All evidence was integrated using the MAKER (v3.00) pipeline [93] to generate a high-confidence, nonredundant set of gene models, which were subsequently evaluated for completeness using BUSCO (v5.2.2) against the Embryophyta_odb10 database. For functional annotation, predicted protein sequences were systematically searched against multiple databases: Swiss-Prot and TrEMBL for protein homology (BLASTP, *E*-value ≤ 1 × 10^−5^), KEGG for metabolic pathways, InterPro for protein domains, gene ontology (GO) for functional classification, and the NR database for comprehensive sequence similarity. This multilayered annotation strategy ensured robust gene prediction and comprehensive functional characterization of the TpraZt5 genome.

A comprehensive pipeline was implemented for the annotation of ncRNA elements using established computational tools and reference databases. tRNAs were identified using tRNAscan-SE (v1.3.1) with default parameters optimized for plant genomes. rRNA annotation was performed through BLASTN (v2.6.0) searches against a curated database of conserved rRNA sequences from related species. For the detection of miRNAs and snRNAs, we employed Infernal (v1.1.2) with covariance models from the Rfam database (v14.1), implementing rigorous *E*-value thresholds (≤0.01) to ensure prediction accuracy. All predicted ncRNAs were further validated through manual inspection of characteristic secondary structures and genomic context, with particular attention to conserved motifs and flanking sequences typical of functional ncRNAs in *Trifolium* genus plants.

### Genome evolution and WGD analysis

To elucidate the evolutionary history of TpraZt5, its phylogenetic positioning was assessed using 14 Fabaceae genomes including four *Trifolium* genus taxa (*T. pratense*, *T. repens* (diploid), *T. subterraneum*, and *T. medium*). Orthologous gene clusters were identified across all species using OrthoFinder (v2.4.0) with default parameters [94]. Single-copy orthologous genes were aligned using MUSCLE (v3.8.31) and the best fit substitution model was selected based on the supermatrix alignment [95]. A maximum likelihood phylogenetic tree was constructed using RAxML (v8.2.12) with 1000 bootstrap replicates, using *Lupinus angustifolius* as the outgroup [96]. Divergence times were estimated using MCMCTREE (v1.0) and calibrated with nine key fossil nodes from the TimeTree database [97], as illustrated in Fig. S13. Gene family expansion and contraction analyses were performed using CAFE (v2.0) [98]. Functional enrichment analyses of significantly changing gene families were conducted using GO and KEGG pathway databases with a significance threshold of *P* <0 .05. Whole-genome synteny analyses were performed using the JCVI toolkit with default parameters [99]. To investigate polyploidization events, we calculated *Ks* for paralogous gene pairs using the PAML package [100]. The *Ks* distributions were analyzed to identify potential WGD events in the evolutionary history of TpraZt5.

### Pan-genome construction

Whole-genome alignments of three *T. pratense* accessions were performed using MUMmer (v4.0) [101]. The pan-genome was subsequently constructed using ppsPCP [102]. Protein sequences from these accessions were clustered into orthogroups using OrthoFinder (v2.3.7) [94], employing Diamond for sequence alignments with a stringent *E*-value threshold of 1 × 10^−3^. Functional annotation of core, dispensable, and private gene families was conducted using the PANTHER (v15) classification system [103]. Finally, clusterProfiler (v3.14.0) was used to perform GO term and KEGG pathway enrichment analyses [104] based on the annotated gene sets to elucidate their biological significance.

### SV identification

To build a genetic variance atlas for the three red clover genomes, we aligned TpraHA7 and TpraMiv genome assemblies to the TpraZt5 reference genome using MUMmer (v4.0), respectively. The alignment of the genomes was performed using nucmer (--maxmatch -c 250 -l 40 -t 6) and the alignment block filter was implemented using a delta-filter program (-1 -i 90 -l 500). Pairwise alignment coordinates between the reference and query genomes were processed using show-coords (-THrd) to generate normalized positional mappings. SyRI (v1.6) [105] was employed to detect genomic variations including TRANS, INV, DUP, CNV, and PAV. Variations were classified as ‘Presence’ when the sequence longer than 50 bp was absent in the reference genome but present in the query genome. Conversely, ‘Absence’ variants were defined as sequences longer than 50 bp exclusively present in the reference genome but missing in the query genome.

## Ethics approval and consent to participate

No ethical approval or permission is required to obtain the materials and perform the research in this study.

## Supplementary Material

Web_Material_uhag013

## Data Availability

The genome sequencing data, including PacBio HiFi, ONT Ultra-long, and Hi-C data, have been deposited into the NCBI database and are available via the BioProject accession number PRJNA1332894. All additional supporting data are available in the supplementary materials.
