# Supplementary material for: A telomere-to-telomere gap-free genome of the new cultivar ‘Zhongtian No. 5’, combined with pan-genome analysis, aids in exploration and genetic enhancement of red clover (Trifolium pratense L.)
Source: Hortic Res. 2026 Jan 13;13(4):uhag013. doi: 10.1093/hr/uhag013 (PMC13103476; doi:10.1093/hr/uhag013)
Supplement: Web_Material_uhag013 [file web_material_uhag013.zip › 11.29-Supplementary Tables .doc]

**Supplementary Table S1.** Statistics of all sequence data of ZhongTian No. 5.

| Library | Organ | Read No. | Base  (G bp) | Coverage (x) | N50  (bp) | Average length (bp) |
| --- | --- | --- | --- | --- | --- | --- |
| Nanopore raw | shoot | 4,440,090 | 91.08 | 233 | 71,936 | 20,513.48 |
| Nanopore clean | shoot | 3,166,996 | 67.15 | 172 | 74,157 | 21,201.94 |
| Hi-C raw | shoot | 672,776,091*2 | 201.83 | 516 | 150 | 150 |
| Hi-C clean | shoot | 659,734,318*2 | 196.42 | 502 | 150 | 150 |
| HiFi | shoot | 1,221,106 | 25.80 | 66 | 21,299 | 21,132.4 |

**Supplementary Table S2.** Statistics of the final assembly of ZhongTian No. 5 genome using HiFi and ONT reads after scaffolding.

| Metrics | Length (bp) | Count |
| --- | --- | --- |
| N10 | 68,907,478 | 1 |
| N20 | 62,836,184 | 2 |
| N30 | 62,836,184 | 2 |
| N40 | 61,203,564 | 3 |
| N50 | 52,947,529 | 4 |
| N60 | 52,947,529 | 4 |
| N70 | 50,460,597 | 5 |
| N80 | 49,753,863 | 6 |
| N90 | 44,828,903 | 7 |
| Total length | 390,938,118 | 7 |
| Max_length | 68,907,478 |  |

**Supplementary Table S3.** Statistics of the telomere-to-telomere (T2T) genome assembly of ZhongTian No. 5.

| Chromosome | Contig No. | Gap No. | Telomere No. | Size (bp) |
| --- | --- | --- | --- | --- |
| Chr1 | 1 | 0 | 2 | 49753863 |
| Chr2 | 1 | 0 | 2 | 68907478 |
| Chr3 | 1 | 0 | 2 | 52947529 |
| Chr4 | 1 | 0 | 2 | 61203564 |
| Chr5 | 1 | 0 | 2 | 62836184 |
| Chr6 | 1 | 0 | 2 | 44828903 |
| Chr7 | 1 | 0 | 2 | 50460597 |

**Supplementary Table S4.** Statistics of telomere sequences in the T2T genome assembly of ZhongTian No. 5.

| Chromosome | Head | | | Tail | | |
| --- | --- | --- | --- | --- | --- | --- |
|  | Start | End | Length (bp) | Start | End | Length (bp) |
| Chr1 | 1 | 3944 | 3.94 | 49748268 | 49753863 | 5.59 |
| Chr2 | 1 | 14153 | 14.15 | 68902438 | 68907478 | 5.04 |
| Chr3 | 1 | 3216 | 3.22 | 52942437 | 52947529 | 5.09 |
| Chr4 | 1 | 5825 | 5.83 | 61199067 | 61203564 | 4.5 |
| Chr5 | 1 | 10572 | 10.57 | 62789451 | 62836184 | 46.73 |
| Chr6 | 1 | 2314 | 2.31 | 44824059 | 44828903 | 4.84 |
| Chr7 | 1 | 8302 | 8.3 | 50459324 | 50460597 | 1.27 |

**Supplementary Table S5.** Statistics of candidate centromere regions in the ZhongTian No. 5 genome assembly.

| Chromosome | Centromere regions | | |
| --- | --- | --- | --- |
|  | Start | End | Length (Mb) |
| Chr1 | 23336565 | 23450469 | 0.114 |
| Chr2 | 34218699 | 34535572 | 0.317 |
| Chr3 | 24462111 | 24501216 | 0.039 |
| Chr4 | 35365894 | 35493355 | 0.127 |
| Chr5 | 23174672 | 23758296 | 0.584 |
| Chr6 | 13609041 | 13865275 | 0.256 |
| Chr7 | 11664763 | 11699038 | 0.034 |

**Supplementary Table S6.** Statistics of repetitive sequences of the ZhongTian No. 5 genome.

| Type | Class | Count | Length of masked  genome (bp) | ratio of masked genome |
| --- | --- | --- | --- | --- |
| LTR | *Copia* | 77671 | 54492249 | 13.94 |
|  | *Gypsy* | 42531 | 35529989 | 9.09 |
|  | Unknown | 206669 | 62855868 | 16.08 |
| TIR | *PIF_Harbinger* | 9941 | 2160470 | 0.55 |
|  | *Tcl_Mariner* | 1074 | 73267 | 0.02 |
|  | *hAT* | 24497 | 5754549 | 1.47 |
|  | *CMC* | 75943 | 16876546 | 4.32 |
|  | *MULE* | 13227 | 3683526 | 0.94 |
|  | Unknown | 19575 | 3114650 | 0.80 |
| non TIR | *Helitron* | 17171 | 5557402 | 1.42 |
| others |  | 509112 | 34337967 | 8.78 |
| Total interspersed |  | 997411 | 224436483 | 57.41 |
| Tandem repeats |  | 180116 | 23626146 | 6.04 |
| Total repeats |  | 1177527 | 232892231 | 59.57 |

**Supplementary Table S7.** Statistics of protein-coding genes in the ZhongTian No. 5 genome assembly.

| Metrics | Gene number | Percentage (%) |
| --- | --- | --- |
| Protein-coding | 35971 |  |
| Interproscan | 25688 | 71.41 |
| GO | 22184 | 61.67 |
| KEGG_all | 33212 | 92.33 |
| KEGG_KO | 11264 | 31.31 |
| Swiss-prot | 24036 | 66.82 |
| TrEMBL | 35124 | 97.65 |
| NR | 34975 | 97.23 |
| Annotated | 35179 | 97.80 |
| Unannotated | 792 | 2.20 |

**Supplementary Table S8.** Quality assessment of the gene set of ZhongTian No. 5 genome using BUSCOs.

| Metrics | Assembly | | Annotation | | |
| --- | --- | --- | --- | --- | --- |
|  | Number | Percentage (%) | Number | | Percentage(%) |
| Complete BUSCOs | 1582 | 98.0 | 1580 | 97.9 | |
| Complete and single-copy BUSCOs | 1516 | 93.9 | 1506 | 93.3 | |
| Complete and duplicated BUSCOs | 66 | 4.1 | 74 | 4.6 | |
| Fragmented BUSCOs | 14 | 0.9 | 14 | 0.9 | |
| Missing BUSCOs | 18 | 1.1 | 20 | 1.2 | |
| Total BUSCO groups searched | 1614 | 100.0 | 1614 | 100.0 | |

**Supplementary Table S9.** Statistics of noncoding RNAs in the ZhongTian No. 5 genome.

| Type | | Copy | Average length (bp) | Total length (bp) | % of genome |
| --- | --- | --- | --- | --- | --- |
| miRNA |  | 124 | 121.55 | 15072 | 0.0039 |
| tRNA |  | 877 | 74.77 | 65577 | 0.0168 |
| rRNA | rRNA | 917 | 2930.43 | 2687201 | 0.6874 |
|  | 18S | 455 | 1824.94 | 830347 | 0.2124 |
|  | 28S | 455 | 4079.26 | 1856064 | 0.4748 |
|  | 5S | 7 | 112.86 | 790 | 0.0002 |
| snRNA | snRNA | 580 | 126.87 | 73586 | 0.0188 |
|  | CD-box | 250 | 103.58 | 25895 | 0.0066 |
|  | HACA-box | 33 | 125.64 | 4146 | 0.0011 |
|  | splicing | 297 | 146.62 | 43545 | 0.0111 |
|  | scaRNA | 0 | 0.00 | 0 | 0.0000 |

**Supplementary Table S10.** Information of 15 representative plant species used in the study.

| Plant species | Genus | Family | Order | The website link of the reference genomic database |
| --- | --- | --- | --- | --- |
| *Lupinus angustifolius* | Lupinus | Fabaceae | Fabales | https://ftp.ncbi.nlm.nih.gov/genomes/all/GCA/001/865/875/GCA_001865875.1_LupAngTanjil_v1.0/GCA_001865875.1_LupAngTanjil_v1.0_genomic.fna.gz |
| *Phaseolus vulgaris* | *Phaseolus* | Fabaceae | Fabales | https://ftp.ncbi.nlm.nih.gov/genomes/all/GCA/000/499/845/GCA_000499845.2_P._vulgaris_v2.0/GCA_000499845.2_P._vulgaris_v2.0_genomic.fna.gz |
| *Glycine soja* | *Glycine* | Fabaceae | Fabales | https://ftp.ncbi.nlm.nih.gov/genomes/all/GCA/004/193/775/GCA_004193775.2_ASM419377v2/GCA_004193775.2_ASM419377v2_genomic.fna.gz |
| *Cicer arietinum* | *Cicer* | Fabaceae | Fabales | https://ftp.ncbi.nlm.nih.gov/genomes/all/GCF/000/331/145/GCF_000331145.1_ASM33114v1/GCF_000331145.1_ASM33114v1_genomic.fna.gz |
| *Medicago truncatula* | *Medicago* | Fabaceae | Fabales | https://ftp.ncbi.nlm.nih.gov/genomes/all/GCF/000/219/495/GCF_000219495.3_MedtrA17_4.0/GCF_000219495.3_MedtrA17_4.0_genomic.fna.gz |
| *Medicago sativa* | *Medicago* | Fabaceae | Fabales | https://figshare.com/articles/dataset/Medicago_sativa_genome_and_annotation_files/12623960 |
| *Trifolium repens a* | *Trifolium* | Fabaceae | Fabales | https://ftp.ncbi.nlm.nih.gov/genomes/all/GCA/030/408/175/GCA_030408175.1_UTM_Trep_v1.0/GCA_030408175.1_UTM_Trep_v1.0_genomic.fna.gz |
| *Trifolium repens b* | *Trifolium* | Fabaceae | Fabales | https://ftp.ncbi.nlm.nih.gov/genomes/all/GCA/030/408/175/GCA_030408175.1_UTM_Trep_v1.0/GCA_030408175.1_UTM_Trep_v1.0_genomic.fna.gz |
| *Trifolium subterraneum* | *Trifolium* | Fabaceae | Fabales | https://ftp.ncbi.nlm.nih.gov/genomes/all/GCA/001/742/945/GCA_001742945.1_TSUd_r1.1/GCA_001742945.1_TSUd_r1.1_genomic.fna.gz |
| *Trifolium medium* | *Trifolium* | Fabaceae | Fabales | https://ftp.ncbi.nlm.nih.gov/genomes/all/GCA/003/490/085/GCA_003490085.1_ASM349008v1/GCA_003490085.1_ASM349008v1_genomic.fna.gz |
| *Trifolium pratense* | *Trifolium* | Fabaceae | Fabales | https://ftp.ncbi.nlm.nih.gov/genomes/all/GCF/020/283/565/GCF_020283565.1_ARS_RC_1.1/GCF_020283565.1_ARS_RC_1.1_genomic.fna.gz |
| *Vicia faba* | *Vicia* | Fabaceae | Fabales | https://ftp.ncbi.nlm.nih.gov/genomes/all/GCA/948/472/305/GCA_948472305.1_Hedin2_genome_v1/GCA_948472305.1_Hedin2_genome_v1_genomic.fna.gz |
| *Vicia villosa* | *Vicia* | Fabaceae | Fabales | https://ftp.ncbi.nlm.nih.gov/genomes/all/GCF/029/867/415/GCF_029867415.1_Vvil1.0/GCF_029867415.1_Vvil1.0_genomic.fna.gz |
| *Pisum sativum* | *Pisum* | Fabaceae | Fabales | https://ftp.ncbi.nlm.nih.gov/genomes/all/GCF/024/323/335/GCF_024323335.1_CAAS_Psat_ZW6_1.0/GCF_024323335.1_CAAS_Psat_ZW6_1.0_genomic.fna.gz |
| *Lathyrus sativus* | *Lathyrus* | Fabaceae | Fabales | https://ftp.ncbi.nlm.nih.gov/genomes/all/GCA/963/859/935/GCA_963859935.3_JIC_Lsat_v2.1.1/GCA_963859935.3_JIC_Lsat_v2.1.1_genomic.fna.gz |
