# Supplementary material for: A telomere-to-telomere gap-free genome of the new cultivar ‘Zhongtian No. 5’, combined with pan-genome analysis, aids in exploration and genetic enhancement of red clover (Trifolium pratense L.)
Source: Hortic Res. 2026 Jan 13;13(4):uhag013. doi: 10.1093/hr/uhag013 (PMC13103476; doi:10.1093/hr/uhag013)
Supplement: Web_Material_uhag013 [file web_material_uhag013.zip › supplementary figures 11.29.doc]

**Supplementary Fig. S1.** Evaluation of Zhongtian No.5 (TpraZt5) genome size by *k*-mer analysis. The x and y axis show *k*-mer coverage depth (×) and frequency, respectively. The frequency of each *k*-mer in raw sequencing reads was calculated for K = 17.

**Supplementary Fig. S2.** The flowchart of assembly for ZhongTian No. 5 (TpraZt5) T2T genome.

**Supplementary Fig. S3.** Peak distribution of centromeric regions on the 7 chromosomes for TpraZt5 genome. The unit of 0-60 is Mb.

**Supplementary Fig. S4.** Statistics of the gene numbers near *Gypsy* LTR-RTs (A) and the KEGG enrichment analysis of these genes (B).

**Supplementary Fig. S5.** Statistics of the gene numbers near *Copia* LTR-RTs (A) and the KEGG enrichment analysis of these genes (B).

**Supplementary Fig. S6.** Statistics of the gene numbers near Unknown LTR-RTs (A) and the KEGG enrichment analysis of these genes (B).

**Supplementary Fig. S7.** Kyoto Encyclopedia of Genes and Genomes (KEGG) enrichment of the expansion genes in the genome of TpraZt5.

**Supplementary Fig. S8.** Kyoto Encyclopedia of Genes and Genomes (KEGG) enrichment of the contraction genes in the genome of TpraZt5.

**Supplementary Fig. S9.** Synteny dot plots among TpraZt5, HEN17-A07 (TpraHA7) [26], and Milvus-BE (TpraMiv, GenBank assembly number: GCA_949352195.3). (A) intraspecies genomic relationships of TpraZt5. (B) Intraspecies genomic relationships of TpraHA7. (C) Intraspecies genomic relationships of TpraMiv. (D) Interspecies genomic relationships between TpraZt5 and TpraHA7. (E) Interspecies genomic relationships between TpraZt5 and TpraMiv. (F) Interspecies genomic relationships between TpraHA7 and TpraMiv.

**Supplementary Fig. S10.** The ratio of non-synonymous to synonymous mutations (Ka/Ks) and gene length in core, dispensable and private genes. The center line represents the median, the box limits represent the upper and lower quartiles.

**Supplementary Fig. S11.** Gene Ontology (GO) enrichment results of core (A), dispensable (B) and unique gene sets (C). Significant differences were tested by hypergeometric test hypergemetric test (*FDR-adjusted *P* < 0.05).

**Supplementary Fig. S12.** Kyoto Encyclopedia of Gnes and Genomes (KEGG) enrichment results of core (A), dispensable (B), unique (C) and TpraZt5 private gene sets (D). Significant differences were tested by hypergeometric test hypergemetric test (*FDR-adjusted *P* < 0.05).

**Supplementary Fig. S13.** Divergence time of the species in phylogenetic tree. The nine red dots in the figure represent the nine key fossil calibration nodes.
